# Supplementary material for: On-chip petahertz electronics for single-shot phase detection
Source: Nat Commun. 2024 Nov 23;15:10179. doi: 10.1038/s41467-024-53788-z (PMC11585632; doi:10.1038/s41467-024-53788-z)
Supplement: Supplementary file 1 — Supplementary Information [file 41467_2024_53788_MOESM1_ESM.pdf]

Supplementary Information for:

On-Chip Petahertz Electronics for Single-Shot Phase Detection

Felix Ritzkowsky[1,2,†,*], Matthew Yeung[2,†], Engjell Bebeti[1], Thomas Gebert[3,4], Toru Matsuyama[3], Matthias Budden[4], Roland E. Mainz[1], Huseyin Cankaya[1], Karl K. Berggren[2,*], Giulio Maria Rossi[1], Phillip D. Keathley[2,*], and Franz X. Kärtner[1,5]

[1]*Center for Free-Electron Laser Science CFEL, Deutsches Elektronen-Synchrotron DESY, Notkestr. 85, 22607 Hamburg, Germany*

[2]*Research Laboratory of Electronics, Massachusetts Institute of Technology, 77 Massachusetts Avenue, Cambridge, MA 02139, USA*

[3] *Max Planck Institute for the Structure and Dynamics of Matter, Luruper Chaussee 149, 22761, Hamburg, Germany*

[4] *WiredSense GmbH, Luruper Hauptstr. 1, 22547 Hamburg, Germany*

[5]*Department of Physics and The Hamburg Centre for Ultrafast Imaging, Universität Hamburg, Luruper Chaussee 149, 22761 Hamburg, Germany*

[†]*These authors contributed equally to this work.*

[*]*e-mail: felix.ritzkowsky@desy.de; pdkeat2@mit.edu*

1. Description of the Sub-Cycle Field Emission Current

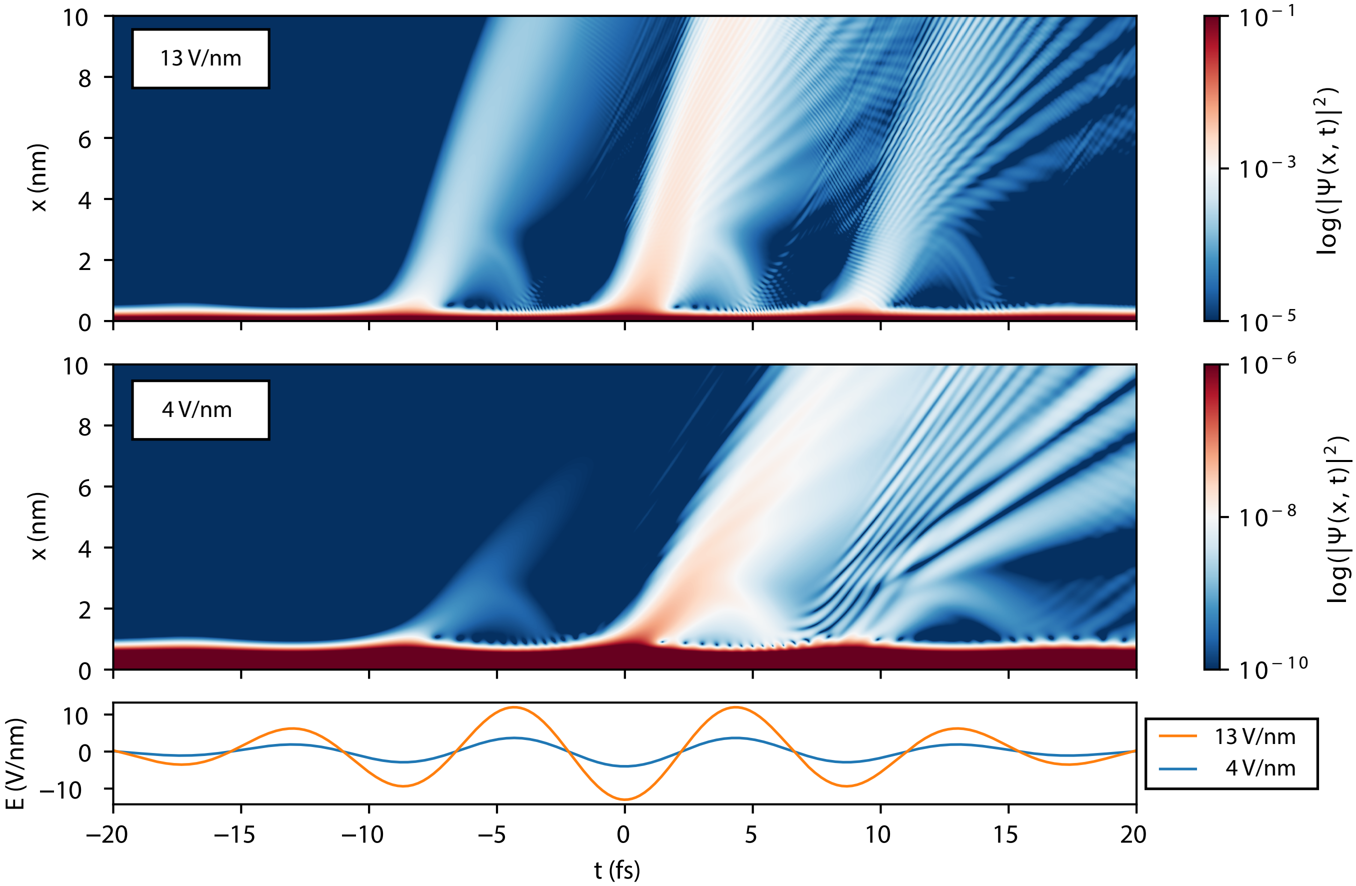

Supplementary Figure 1: **Numerical Solution to the Time-Dependent Schrödinger Equation:** Integration of the time-dependent Schrödinger equation, by using the modified Crank-Nicolson scheme as described by [1]. The top two panels show the probability amplitudes for two cases assuming an 18 fs duration MIR pulse at a center wavelength of 2.7 µm with 4 V nm^{-1} and 13 V nm^{-1} field strength. The bottom panel is showing the electric field waveform as a function of time for each case.

To gain in qualitative understanding in the sub-cycle dynamics of the field emission process, it is useful to simulate the probability of measuring an electron at a given point in time and space outside the metal in a vacuum state. To that end, Yalunin et al. showed that the numerical integration of the time-dependent Schrödinger equation [1],

$$i\frac{\partial \Psi}{\partial t} = \left(-\frac{1}{2}\frac{\partial^2}{\partial x^2} + V\right)\Psi, \tag{1}$$

describing the interaction of a bound electron in a metal with a time-periodic field $F(t)$ is a valid approach.

The potential V used is of the form,

$$V = -\begin{cases} xeF(t), & x \geq 0 \\ E_F + W, & x < 0, \end{cases} \tag{2}$$

were E_F is the Fermi energy and W the workfunction. By using the modified Crank-Nicolson scheme as described in [1], we calculate the probability $|\Psi(x,t)|^2$ for a gold workfunction of $W = 5.1$ eV and an 18 fs duration MIR pulse at a center wavelength of 2.7 µm. The used field strength is $4\,\mathrm{V\,nm^{-1}}$ and $13\,\mathrm{V\,nm^{-1}}$ to reflect the strength of the local fields measured in Fig. 5 in the main text.

The results of the integration, presented in Fig. 1, show the probability $|\Psi(x,t)|^2$ of measuring an electron at a given coordinate (x,t). At the low field strength of $4\,\mathrm{V\,nm^{-1}}$ the emission process is already highly sub-cycle. Driven by the peak of the electric field the electrons are ejected from the surface within a half-cycle. Strong scattering of electrons re-accelerated to the potential barrier occurs between 5 fs and 10 fs. For the case of $14\,\mathrm{V\,nm^{-1}}$ we can see stronger emission probabilities with suppressed quiver motion and rescattering at the surface. Both cases show that for the full field strength range explored in the main text in Fig. 5, the emission mechanism is still sub-cycle field emission.

Despite the fact that such numerical integration qualitatively and quantitatively describes the scaling laws of electron emission [1], we found difficulties adapting this scheme when accounting for our experiment as described in Eq. 3 of the main text. The differential readout scheme leaves only the CEP-dependent charge, which is on the order of 0.1 or less of the total emitted charge. We found in the end that the measured CEP dependent charge yield was better described by the quasi-static Fowler-Nordheim approximation [2], which was used throughout the main text.

2. Electromagnetic Simulation of the Nanoantenna

An electromagnetic simulation was performed to estimate the local field at the apex of the nanoantenna. A simulation procedure similar to the one described in [3] was used. A fully linear response of the device is assumed, which allows for calculation of the response function in the frequency domain. A numerical solution of the Maxwell equations is obtained using the finite element method electromagnetic waves, frequency domain solver from the wave optics module of COMSOL Multiphysics. The system was modeled by a connected antenna bow-tie consisting of gold placed on a glass substrate. The dimensions of the

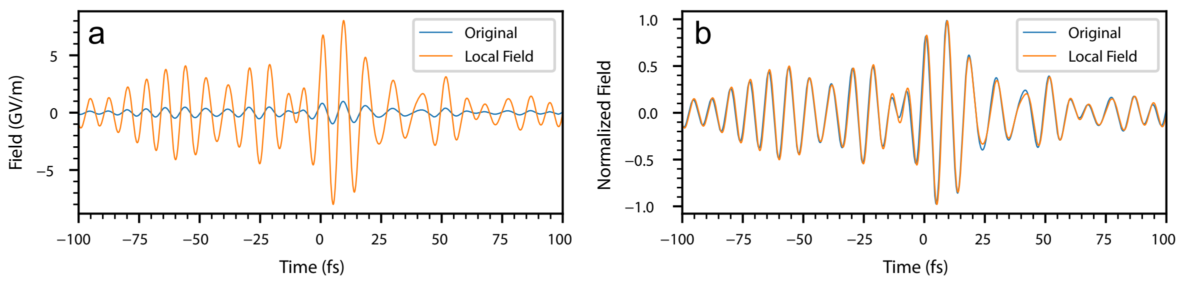

Supplementary Figure 2: **Comparison of the Original Electric Field vs. the Local Field: a,** The original incident electric field waveform and the calculated local field at the tip of the nanoantenna as a function of time. The original field has a peak amplitude of $1\,\mathrm{GV\,m^{-1}}$ and the local field of $8.2\,\mathrm{GV\,m^{-1}}$. **b**, The two field waveforms are normalized to their peak values for a qualitative comparison.

antenna geometry were chosen to fit the fabrication design parameters. The n and k values of gold were taken from [4] and a constant refractive index of 1.46 was assumed for the glass substrate. An incident plane wave with a propagation direction perpendicular to the antenna-substrate interface was added on top of the geometry. The incident light is linearly polarized with the electric field being orthogonal to the connecting wires. Periodic boundary conditions were added around the antenna boundary to model the array. The semi-infinite vacuum and substrate were modeled using perfectly matched layers on the top and bottom of the simulation domain. The linear response function was evaluated by comparing the results obtained with the results of an empty simulation domain with the same simulation settings. To calculate the spatial averaged of the antenna response, we integrated over the whole curvature of the nanoantenna tip, which is assumed to have a radius of curvature of 10 nm and a height of 20 nm.

With the simulated complex frequency response $\tilde{H}(\omega)$, see Fig. 2, and the incident field $E(t)$ the local field $E_{\mathrm{loc}}(t)$ averaged over the surface of the nanoantenna tip can be calculated,

$$\tilde{E}_{\mathrm{loc}}(t) = \mathscr{F}^{-1}\{\tilde{E}(\omega) \cdot \tilde{H}(\omega)\}. \tag{3}$$

The resulting normalized local field is shown in Fig. 2. The local field is only marginally different from the incident electric field. However, the effective field enhancement is 8.2, making the local field substantially stronger than the incident one. Compared to the field enhancements of around 20 in references [3, 5–8], the antenna was designed to be off-resonant to preserve the incident electric field shape, while still having a sufficiently large field enhancement. Furthermore, the antenna design allows for a high antenna density

of $\sim 3\,\mu m^{-2}$, compared to an antenna design that is resonant with the MIR field, since these would require roughly double the antenna size.

2.1. *Geometric Design Study*

The antenna arrays presented in the main text were designed to enable high charge yield with low sensitivity to fabrication errors when fabricated using a robust lithography process flow. Further investigation of the design showed that the maximum value of the CEP-dependent current can be improved by up to a factor of two by increasing the the amount of antennas per unit area. The parameterized antenna unit-cell geometry is shown in Fig. 3 a. The increased density damps the resonant part of the response function but maintains a broadband off-resonant field enhancement with a factor of $\sim 6-7$. The gradual change of antenna density is shown in Fig. 3 b. Using the formulas of the quasi-static model, the increase in CEP-dependent current per unit area is estimated and corresponds to a factor of two improvements from the device presented in the main text.

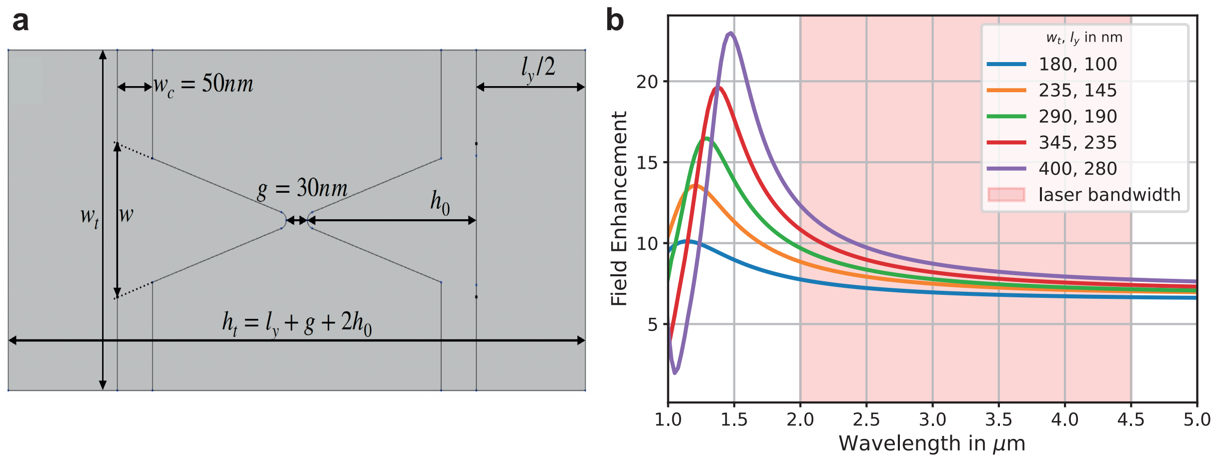

Supplementary Figure 3: **Simulated Field Enhancement for Different Antenna Densities: a** Parameterized antenna geometry. **b,** The average field enhancement at the nanoantenna apex as a function of wavelength for different antenna densities. w_t denotes the distance between neighboring antennas and l_y is the closest distance between two arrays. The values $w_t = 400\,\text{nm}$ and $l_y = 280\,\text{nm}$ correspond to the fabricated device presented in the main text. The densest case with $w_t = 180\,\text{nm}$ and $l_y = 100\,\text{nm}$ has an approximately 2.9 times higher antenna density while maintaining a similar off-resonant field enhancement.

However, by not only optimizing the network density, but also adjusting the resonance of the field en-

hancement while still ensuring off-resonant excitation, we can achieve further improvements in the generated CEP dependent current. The results of this optimization is shown in Fig. 4.

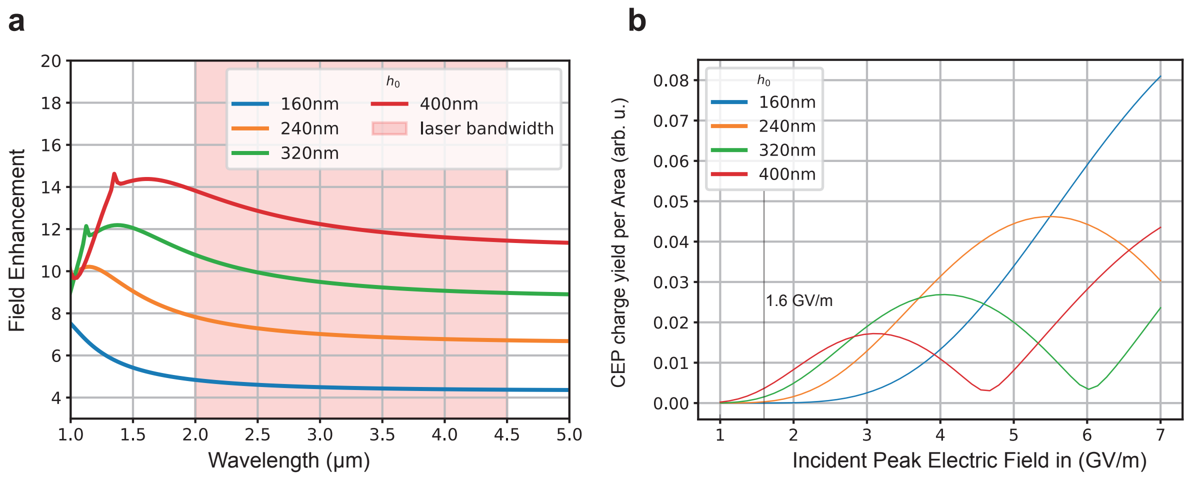

Supplementary Figure 4: **Simulated Field Enhancement for Different Antenna Resonances with the Optimum Density: a,** The average field enhancement at the nanoantenna apex as a function of wavelength for different antenna configurations, varying the antenna height h_0 with a fixed width at a ratio of $w = 3/4h_0$, while keeping the most dense configuration of the network. **b**, CEP sensitive current normalized to the unit area for the different antenna variations in a.

The idea of this study is to sweep the resonance of the antenna length at the most dense network configuration. The resonance is mostly defined by the antenna height h_0, with a fixed width of the antenna base of $w = 3/4h_0$. The unit-cell dimension is chosen at $w_t = w$ an the horizontal antenna spacing at l_y =100 nm yielding a cell width of $h_t = l_y + g + 2h_0$, where g denotes the gap width of 30 nm. By calculating the CEP-dependent charge yield per individual antenna and normalized to the occupied area of the unit cell, we can define the normalized CEP sensitivity and effectively compare the different configurations. The normalized CEP sensitivity is plotted in Fig. 4. For a given operating point for the peak electric-field of the excitation pulse, we can find the optimum configuration. At 1.6 GV m^{-1}, we find that an antenna height of 400 nm gives up to 7-fold improvement in CEP sensitive current, compared to what was tested in the main text. With this naive optimization process we can already see that substantial improvements in antenna design can be found. Further study of the multidimensional parameter space, will certainly allow to find the actual global maximum for the antenna design.

3. Experimental Setup

3.1. Laser Source Characterization

The laser source used to run the experiments is described in detail in reference [9]. But to reflect the state of the laser source used for the described experiment, we show here the characterization of the pulse duration within a 24 h time window to the experiment. The pulse duration is measured by an adapted version of two-dimensional spectral shearing interferometry (2DSI) [10, 11], where the ancillary pulses are derived from the pump laser at 1.03 µm instead of directly from the mid-infrared pulse under test[9]. This allows for the use of broadly available and cost-effective spectrometers based on Si detector arrays, since in this implementation the up-converted spectrum will cover the NIR from 600 nm to 900 nm. The ancillary pulses are generated by fine-tuned narrowband line filters in a Michelson interferometer, resulting in a shear frequency of 1.35 THz. The resulting measurement is shown in Fig. 5 in logarithmic color coding. The retrieved group delay is shown on the right-hand y-axis. As can be seen, the group delay is reasonably flat and shows a sharp oscillation at 2.7 µm, which corresponds to known water absorption lines [12].

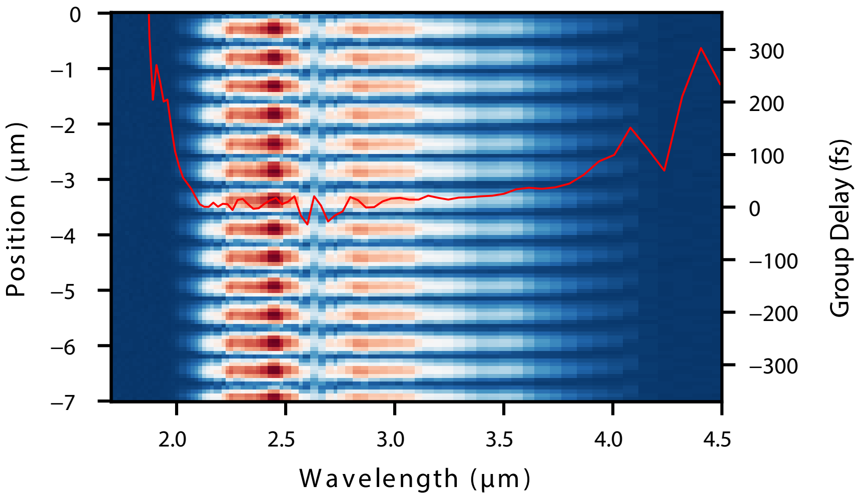

Supplementary Figure 5: **Two-Dimensional Spectral Shearing Interferometry:** Measured 2DSI trace shown in logarithmic color coding. On the right-hand axis in red is an overlay of the retrieved group delay.

In Fig. 6 the raw mid-infrared spectrum is shown. The spectrum was measured on a PbSe-CCD spectrometer. Using the measured mid-infrared spectrum and the retrieved group delay, the time domain of the pulse can be calculated up to an arbitrary CEP. The calculated time domain in intensity and electric field

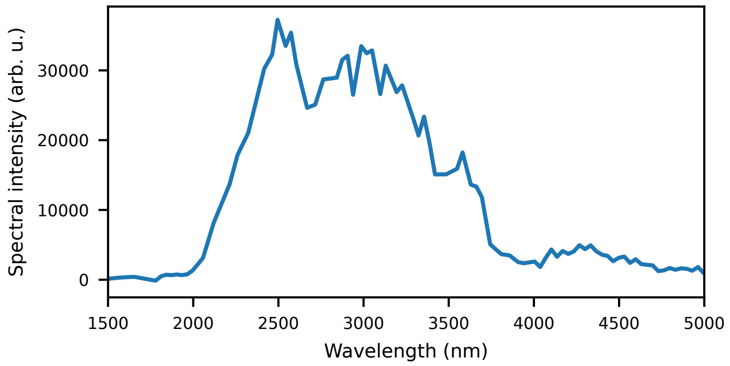

Supplementary Figure 6: **Measured Mid-Infrared Spectrum:** Raw mid-infrared spectrum measured on a PbSe-CCD spectrometer.

are shown in Fig. 7. The retrieved pulse FWHM duration is 18 fs at a center wavelength of 2.69 µm. This corresponds to two cycles of the carrier wave within the FWHM duration.

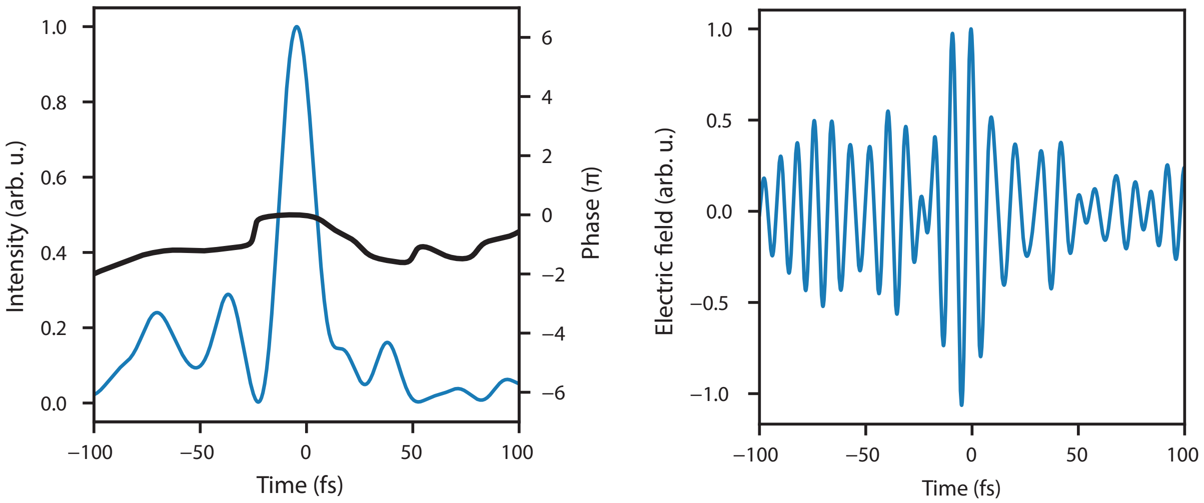

Supplementary Figure 7: **Reconstructed Time Domain:** (Left) Reconstructed intensity distribution of the mid-infrared pulse (blue) and the retrieved phase (black). (Right) Electric field profile of the retrieved mid-infrared pulse set at an arbitrary CEP.)

The passive CEP stability of the system was measured with f-$2f$ interferometry to be lower than 190 rad RMS, with details found in Ref. [9]. The CEP was controlled by adjustment of the pump-seed delay of the adiabatic difference frequency generation stage. To move the CEP by 2π of the mid-infrared pulse, the pump is delayed by one wavelength λ =1.03 µm. The delay is produced by a slip-stick piezo stage (SLC-2430, Smaract GmbH) and a hollow roof mirror, which delays the pump.

3.2. Measurement of Focal-Spot Size

To have an accurate estimation of the peak intensity in our experiment we used the knife-scan technique to measure the beam-size of the MIR beam inside the focus. To realize a sharp edge, we used the lithographically defined leads of the chip itself, as seen in Fig. 14 and scanned the edge through the focus for the horizontal and vertical dimension with 1 µm and 2 µm step-size, while measuring the transmitted intensity with a pyro-electric detector.

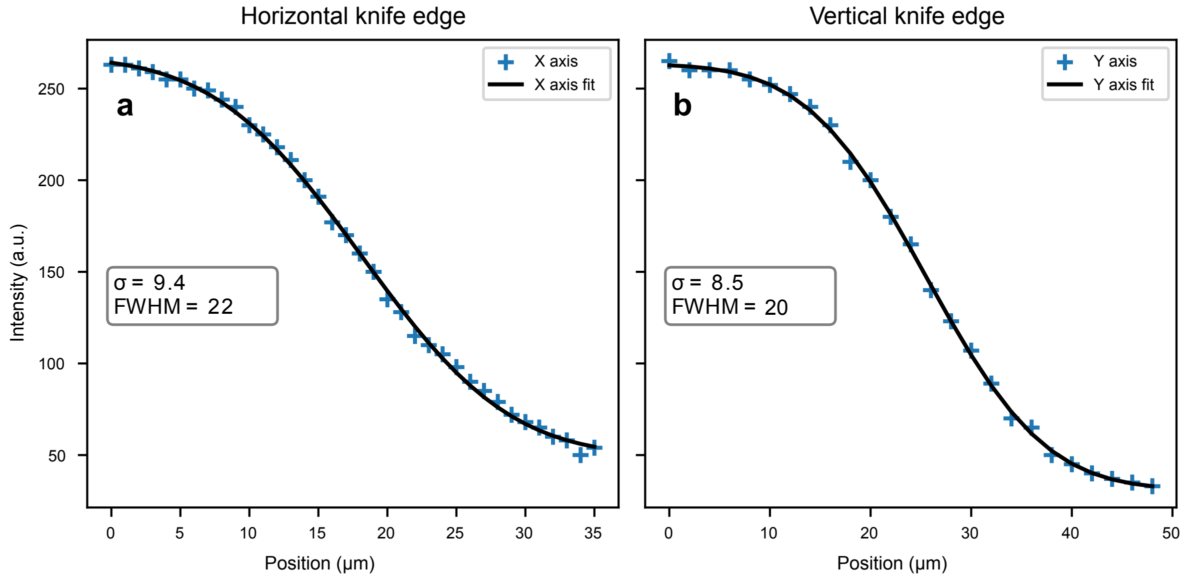

Supplementary Figure 8: **Focal-Size Measurement: a,** Horizontal knife-edge measurement with a resolution of 1 µm. The error-function fit results in a FWHM of 22 µm. **b**, Vertical knife-edge measurement with a resolution of 2 µm. The error-function fit results in a FWHM of 20 µm.

The measured intensity as a function of position is shown for the horizontal and vertical beam-axis in Fig. 8. To retrieve the FWHM of the beam axis, we used a fit with the error-function $\mathrm{erf}(x)$,

$$P(x) = P_{\mathrm{Min}} + (P_{\mathrm{Max}}/2) * (\mathrm{erf}((x - x_0)/(\sigma \cdot \sqrt{2}))). \tag{4}$$

The fit parameters are the minimum intensity P_{Min}, the maximum intensity P_{Max}, the center position x_0 and the standard deviation σ. The resulting parameters are $\sigma_{\mathrm{Horiz.}} = 9.4$ and $\sigma_{\mathrm{Vert.}} = 8.5$, which correspond to the FWHM with $\mathrm{FWHM} = 2\sqrt{2\log(2)} \cdot \sigma$. Giving for the horizontal axis a FWHM of 22 µm and for the vertical FWHM 20 µm. Qualitatively the focal-spot shape is well approximated by a gaussian intensity distribution, as indicated by the fit of the error-function.

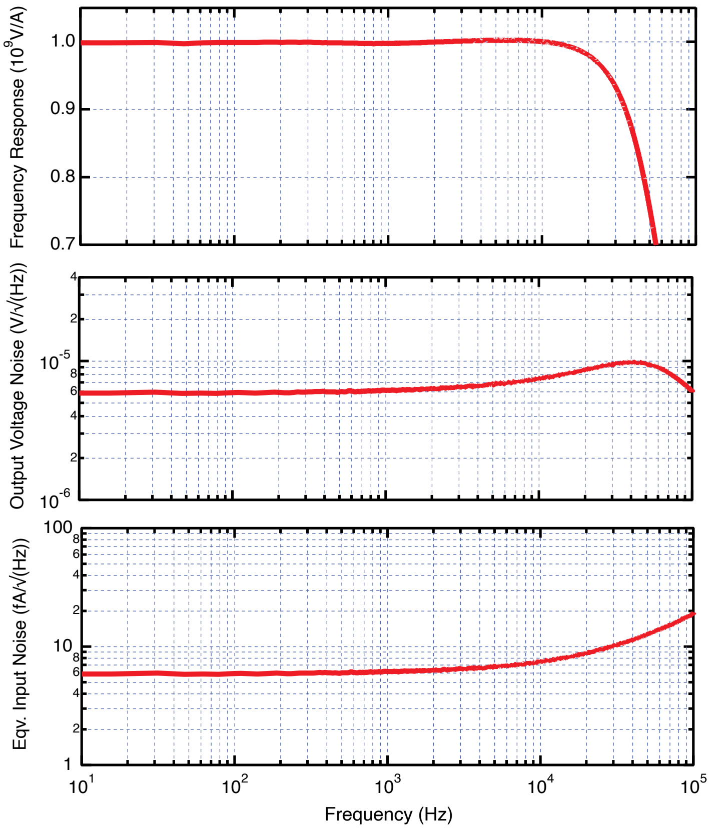

Supplementary Figure 9: **Characterization of the Transimpedance Amplifier:** The TIA is characterized by measurements with an FFT analyzer. (Top panel) The frequency response curve of the TIA. (Middle panel) Measured output voltage noise. (Bottom panel) Equivalent input current noise, is calculated by dividing the output voltage noise by the frequency response of the TIA.

For the readout of the charge in the nanoantenna network, we used a custom trans-impedance amplifier (TIA) provided by WiredSense GmbH. The amplifier has a total gain of $1\,\mathrm{GV\,A^{-1}}$ at a $-3\,\mathrm{dB}$-bandwidth of 50 kHz. The input and the output of the TIA are AC coupled. The full response function, the output voltage noise, and the equivalent input noise of the TIA are shown in Fig. 9. The pyroelectric detector used to detect the shot-to-shot intensity changes was also provided by WiredSense GmbH and its frequency response, noise equivalent input power, and detectivity are shown in Fig. 10. The detector was characterized by a calibrated black body radiator. To digitize both signal channels and retrieve the individual charge yields, we used an 8-bit oscilloscope recording the TIA, the pyroelectric detector, and a trigger signal provided by the laser source at a sampling rate of 10 MSa/s. For the 50 kHz repetition rate signal, the sampling rate was chosen to provide

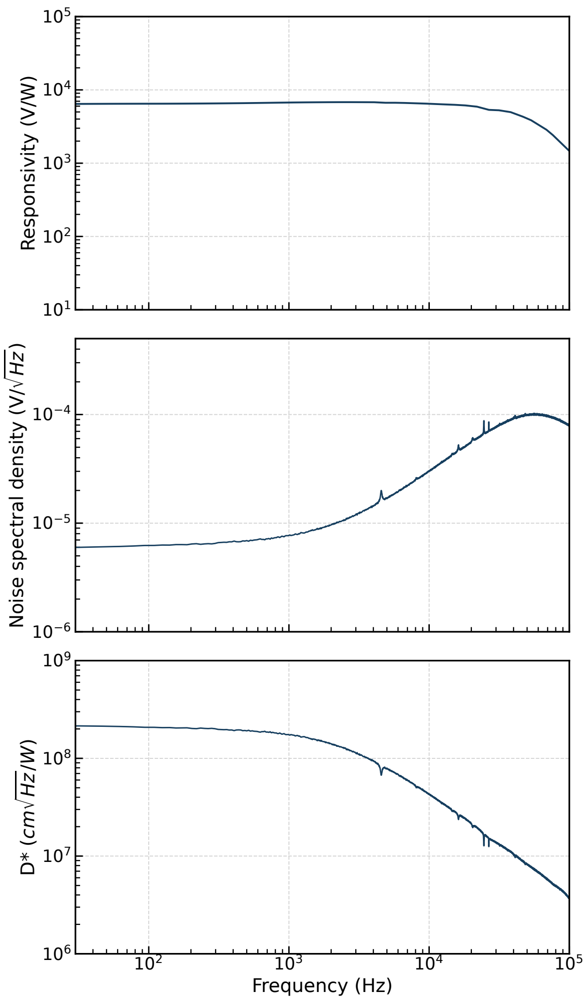

Supplementary Figure 10: **Characterization of the Pyroelectric Detector:** The detector is characterized by measurement with an FFT analyzer and a calibrated black body radiator at 150 °C. (Top panel) The frequency response of the detector. (Middle panel) Noise spectral density of the detector with the blocked input signal. (Bottom panel) Detectivity of the detector.

sufficient oversampling to alleviate digitization problems of the low bit-rate oscilloscope. Oversampling by a factor of x and integration of a digital signal increases the effective bit-range n by $n = \ln(x)/(2\ln(2))$ if the lowest bit is submitted to sufficient Gaussian noise. An example of a recorded oscilloscope trace is shown in Fig. 11

The trigger signal falling slope was used as a reference to sort the individual shots with their respective time stamp. Before integrating over the AC coupled current signals, a baseline was introduced by averaging over the signal for 3 μs before every trigger and subtracting it locally for the respective time windows. The result of that subtraction is shown in Fig. 12. To retrieve the charge contained within each current pulse,

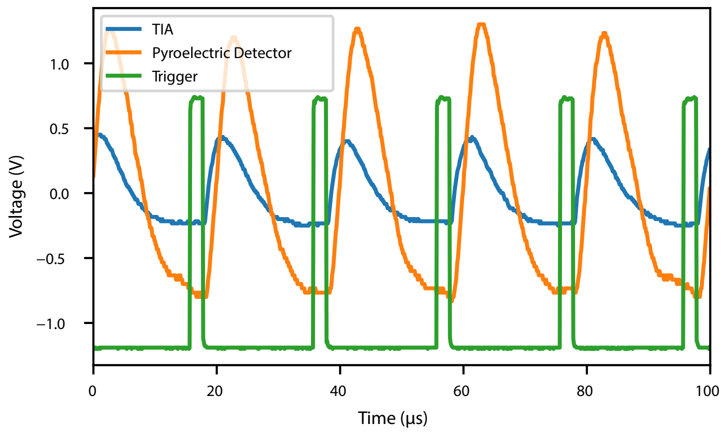

Supplementary Figure 11: **Recorded Oscilloscope Trace:** Exemplary recorded 100 μs voltage time trace showing the trigger signal (green), the TIA output (blue), and the pyroelectric detector output (orange).

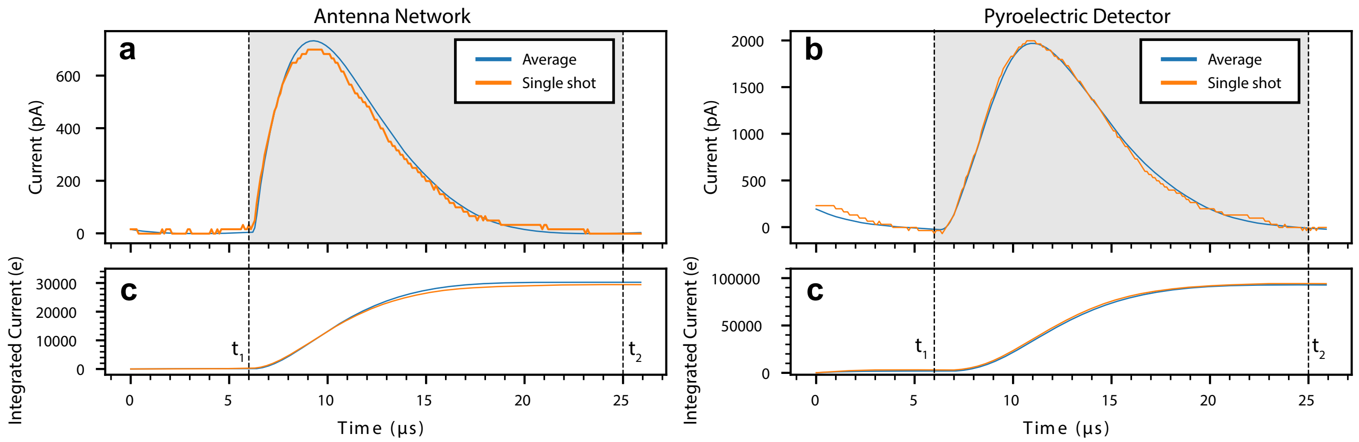

Supplementary Figure 12: **Charge Integration Scheme:** (**a**) Antenna network current pulse, shown for a single pulse and averaged over many pulses to reduce noise and digitization errors. (**b**) Pyroelectric detector current pulse, showing a single pulse and the averaged pulse form. (**c**) The integrated current of the antenna network as a function of time shows a single shot and the averaged pulse form. t_1, t_2 denotes the sampling points used for the charge calculation. (**d**) The integrated current of the pyroelectric detector pulse shows a single shot and the averaged pulse form. t_1, t_2 denotes the sampling points used for the charge calculation.

the current pulse was integrated over and the integrated signal is sampled at points t_1, t_2. The integrated charge is simply the difference of charges measured at the sampling points, $Q_{Shot} = Q(t_2) - Q(t_1)$. This technique is called correlated double sampling (CDS) and is commonly used in charged-coupled device readout circuitry[13]. The time-correlated differentiation significantly reduces uncorrelated low frequency noise[14].

3.4. *Device Layout*

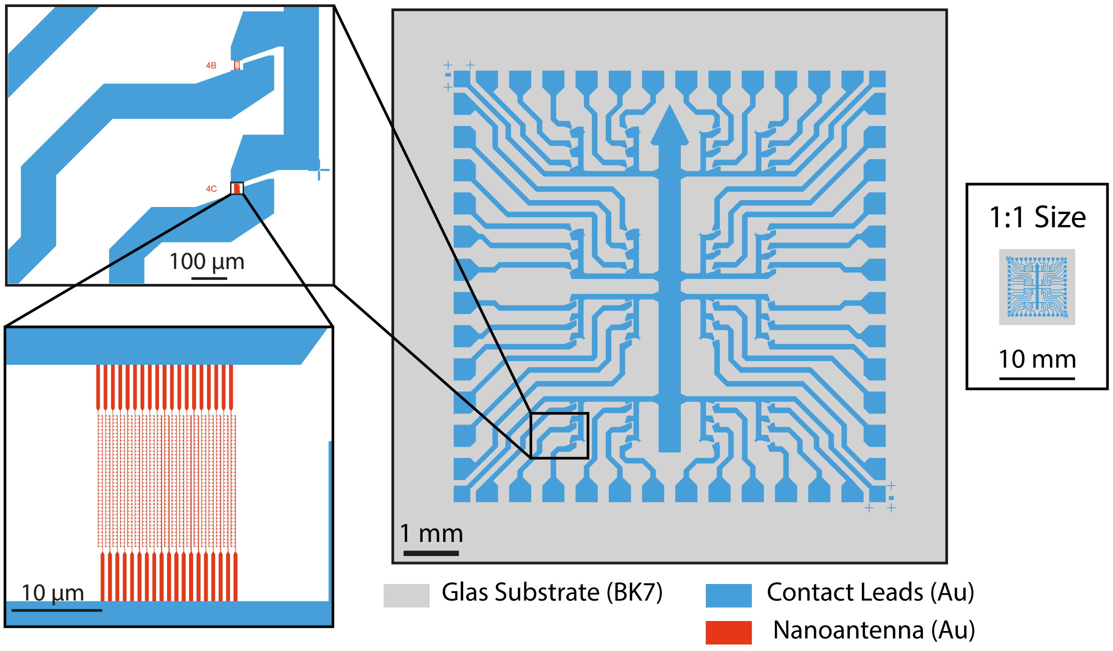

Supplementary Figure 13: **Circuit Layout:** Layout of the microchip carrying the nanoantenna arrays. Progression of sizes from a 1:1 scale down to 1:1000 (Assuming Din-A4 printout of this page). Blue areas mark the contact leads fabricated through photolithography in gold (Au). Red areas mark the nanoantenna arrays fabricated through electron-beam lithography in gold (Au). Grey area marks the glas (BK7) substrate.

Fig. 13 shows the complete layout of the tested chip that contains the nanoantenna arrays. The chip layout is shown on a 1:1 scale with zoom-in on the relevant array tested in the main text. The device, as stated in the Methods Section *Nanofabrication*, is fabricated through a two-step process. The small-scale nanoantenna arrays are fabricated on a BK7 substrate through electron-beam lithography in gold (Au). The second step is the fabrication of larger-scale contact leads through photolithography in gold (Au). The role of the contact leads is to make robust electrical contact with the nanoantenna arrays and to provide large pads at the outer edge of the chip for wire bonding. By visual analysis in a scanning electron microscope, the best 24 arrays of 48 are selected for wire bonding to a printed circuit board. The layout of the device presented in the main text is shown in Fig. 13 a. The nanoantenna array itself is shown by the red-shaded structure and is produced through electron-beam lithography. The pink area is showing contact leads that connect the nanoscale devices with the large wire bond pads at the edges of the chip. The device measures 15 µm by 15 µm for the nanoantenna array. For comparison, the spatial dimension of the optical focus (FWHM and

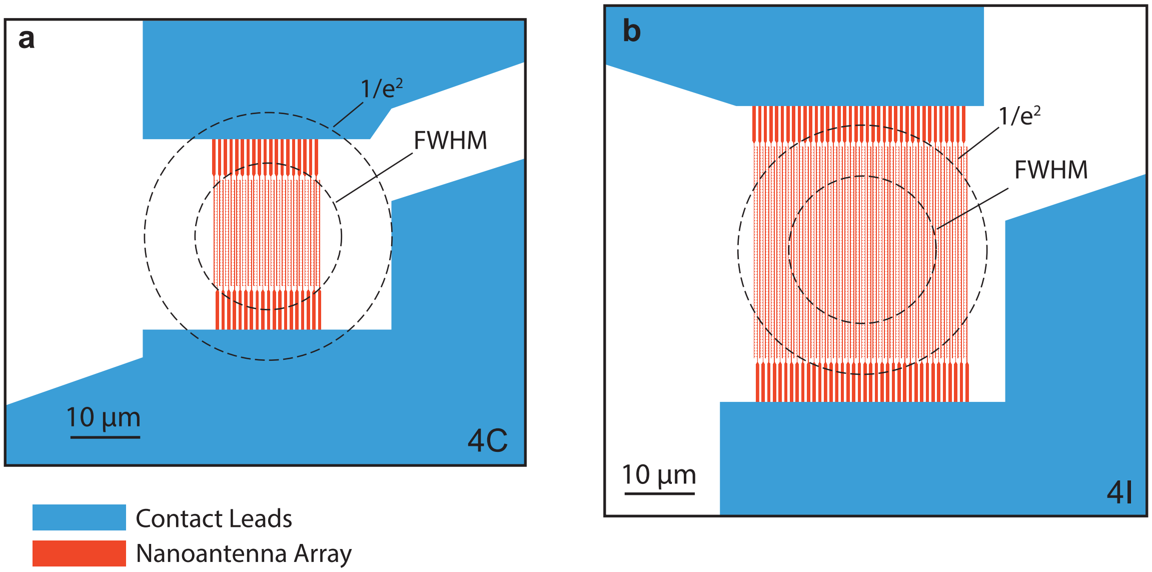

Supplementary Figure 14: **Device Layout:** Layout of the devices used in this work. The blue area shows the contact leads fabricated by photolithography. The red areas show the nanoantennas made by electron-beam lithography. The dashed circles mark the approximate spatial distribution of the laser focus (FWHM: 21 µm, $1/e^2$: 35.5 µm). (**a**) The layout of the device is shown in the main text. The nanoantenna array area of the device measures 15 µm by 15 µm. (**b**) The layout of the device shown in the supplementary. The nanoantenna array area of the device measures 30 µm by 30 µm.

$1/e^2$) is shown as dashed circles. An additional device with an area of 30 µm by 30 µm is shown in Fig. 14 b. The two devices show the case of an array smaller than the laser focus and larger than the laser focus.

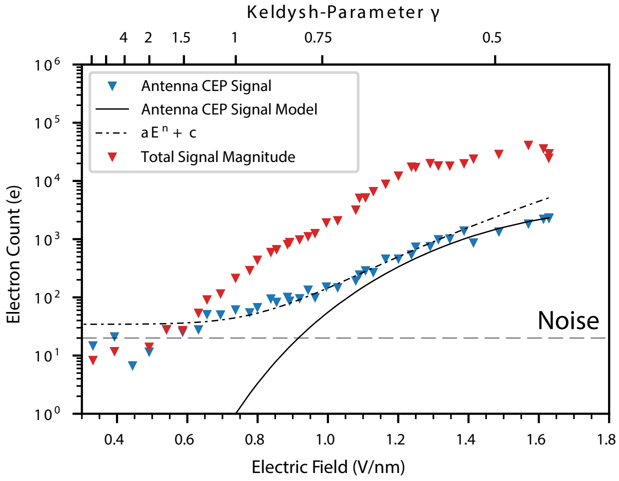

Supplementary Figure 15: **Charge yield scaling:** The CEP modulation amplitude and the total magnitude (average of each measurement) of the electron counts are plotted as a function of the peak field (average of each measurement), estimated for CEP= 0, for the respective dataset. The CEP signal is taken from the amplitude of the 10 Hz frequency component of the measurement data. The antenna CEP signal model uses the model described by Eq. 3 in the main text. The Fowler-Nordheim model describing the tunneling current yields a field enhancement of $g = 7.41$. Furthermore, a power law fit, $aE^n + c$ to the first 30 values is shown.

4. Complementary Measurements

4.1. Field Dependent Scaling of the CEP-Sensitive Charge Yield

To elucidate the mechanism underlying the CEP-dependent electron emission, the amplitude of the CEP modulation is shown in Fig. 15 (blue triangles) as a function of the incident peak field of the laser pulse. In order to estimate the CEP-dependent emission, we first calculated the field at the tip by convolving the applied field (as retrieved by optical pulse characterization) with the calculated impulse response function of the nanoantenna. For peak fields larger than 1.2 V nm^{-1}(estimated for CEP= 0), corresponding to a Keldysh parameter $\gamma \sim 0.6$, the CEP-dependent charge yield scales according to the quasi-static tunneling approximation shown in Eq. 3 (main text). Here our fit model, using the a field-enhancement factor g and a pre-factor α results in a field-enhancement of $g = 7.41$, which is in good agreement with the calculated enhancement 8.2 given the design antenna geometry. Alpha results in $\alpha = 1517$. For values below 1.2 V nm^{-1}, the data follows a power law model $aE^n + c$, with $n = 7.85$, $a = 110$ and $c = 35$. This scaling behavior suggests a transition from nonadiabatic tunneling emission to the quasi-static tunneling regime [15, 16]. This scaling behavior was verified by repeating the experiment with a different nanoantenna array. The results of this second device are presented here in Supplementary Sec. S4.2. The interaction of the optical pulse with our

nanoantenna arrays generates not only CEP-dependent charges but also a pulse energy-dependent charge offset. The magnitude of the average charge yield of each trace (red triangles) is around one order of magnitude larger than the CEP-dependent yield and scales nonlinearly with the pulse energy. It should be noted that this current does not increase monotonically, but goes through a local minimum in the field range from $1.25\,\mathrm{V\,nm^{-1}}$ to $1.6\,\mathrm{V\,nm^{-1}}$. This current scales differently from the CEP-dependent current, implying a different origin than the nanoantenna array. Additional investigation is required as we suspect parasitic field emission from the electrodes close to the nanoantenna array or thermal emission processes plays a role. We believe that an improved electrode design would greatly suppress the charge offset. Similar behavior of the charge offset has been observed in a different size antenna network shown here in the supplementary in Sec. 4.2.

4.2. Background Charge Signal

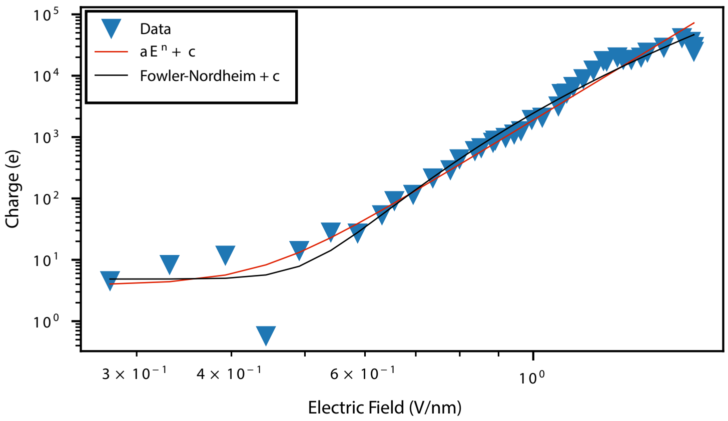

Supplementary Figure 16: **Background Charge Yield:** The average charge yield as a function of peak electric field. A multi-photon absorption and Fowler-Nordheim-based model are fitted to the data. For the multi-photon the function $Q(E,n,\alpha) = \alpha \cdot E^n + c$ is used, with fit results $\alpha = 1.9 \cdot 10^3$, $n = 7.4$ and $c = 3.9$. For the Fowler-Nordheim fit the function $\Gamma(E,g,\alpha) = \alpha(gE)^2 \exp\left\{\left(-\frac{78.7}{|gE|}\right)\right\} + c$, with the results $\alpha = 1705$, $g = 15.4$ and $c = 4.9$.

During the measurements across all tested devices, we observed an intensity-dependent charge background. To investigate the possible origin of this contribution, we tested two different hypotheses. First, the charge signal is generated by a multiphoton emission process, and second that it is generated by a field emission process based on Fowler-Nordheim tunneling. The results are shown in Fig. 16. The multiphoton

fit is defined as,

$$Q(E,n,\alpha) = \alpha \cdot (E)^n + c, \tag{5}$$

with the polynomial order n and a scaling prefactor α. $n = 7.4$, $\alpha = 1.9 \cdot 10^3$ and the offset $c = 3.9$. This hypothesis implies that a 3-4 photon process is causing electron emission, which is incompatible with the photon energy of the optical pulse spanning 0.3 eV to 0.6 eV and the work function of gold with 5.1 eV. This means that either another process is inducing a current other than electron emission from gold or that multiphoton is not the right explanation. The second tested hypothesis is that of a field emitter other than the nanoantenna array. To test this we used the Fowler-Nordheim fit function $\Gamma(E)$,

$$\Gamma(E,g,\alpha) = \alpha (gE)^2 \exp\left\{ \left(-\frac{78.7}{|gE|} \right) \right\} + c, \tag{6}$$

with the prefactor α, the field enhancement g and critical field strength of $78.7\,\mathrm{V\,nm^{-1}}$. The Fit results show a prefactor of $\alpha = 1705$, a field enhancement of 15.4, and the offset $c = 4.9$. This result implies that there is a different field emitter causing this charge yield, as the designed field enhancement of the nanoantenna is on the order of ~ 8. However, as this is merely quantitative speculation, further research is warranted to uncover the cause of this charge contribution. One test experiment could be to use an identical device but excluding the nanoantenna arrays. With that, all contributions from the large gold leads, if also contributing, can be measured independently. Second, the change of polarization could be tested, as these nanoantennas are highly polarization sensitive [8]. However, continuous changing of polarization in the MIR is difficult due to a lack of suitable achromatic waveplates and cannot easily be implemented.

4.3. Estimation of the Spatial Charge-Yield Distribution

Given the measured field dependence in Sec. 4.1, we can estimate the spatial dependence of emitted charge across the whole array. The devices presented in the main text have an array with a size of 15 µm by 15 µm or equivalently 19 by 38 devices that are illuminated with a beam FWHM larger than the array, as also schematically shown in Fig. 14. However, this is only a rough estimation and a more quantitative approach is warranted to understand what fraction of the total array contributes to the measured charge. Based on the focal-spot measurement of 22 µm by 20 µm and combined with the extracted field strength dependence, we can calculate the spatial charge-dependence and normalize it by the amount of antennas.

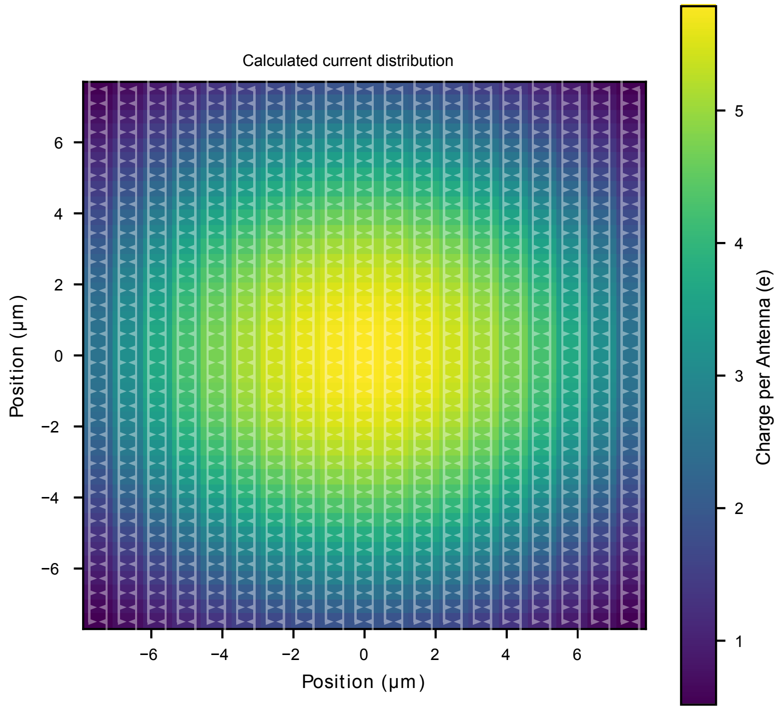

Supplementary Figure 17: **Spatial Dependence of the Charge-Yield:** Heatmap of the spatial charge-yield per antenna normalized to the total measured amount of 2370 e. For reference the schematic of the array is overlayed in grey with accurate scaling.

In Fig. 17 the resulting charge distribution normalized to the total yield of 2370 e is shown. The emitted charge ranges from < 1 e/antenna to close to > 5 e/antenna, with an average yield of 3.3 e/antenna.

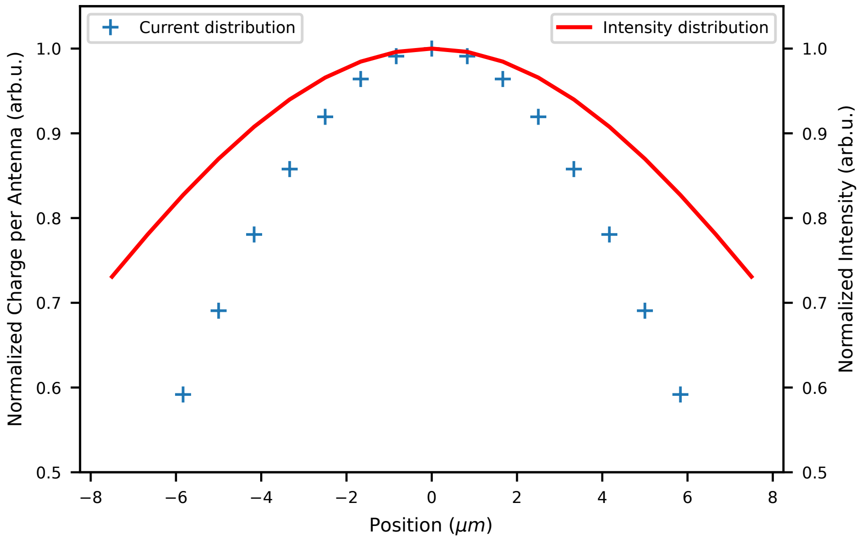

Supplementary Figure 18: **Spatial Dependence of the Charge-Yield along the Horizontal Axis:** 1D-cut of Fig. 17 at 0.8 µm with the normalized charge-yield per antenna and the intensity distribution in red for comparison.

Comparing a horizontal cut of this charge distribution to the intensity distribution of the laser pulse in Fig. 18, we can see that the charge distribution is narrower, as expected due to the nonlinear dependence of the emission process on the peak intensity. The FWHM of the charge yield per antenna is roughly 12 µm.

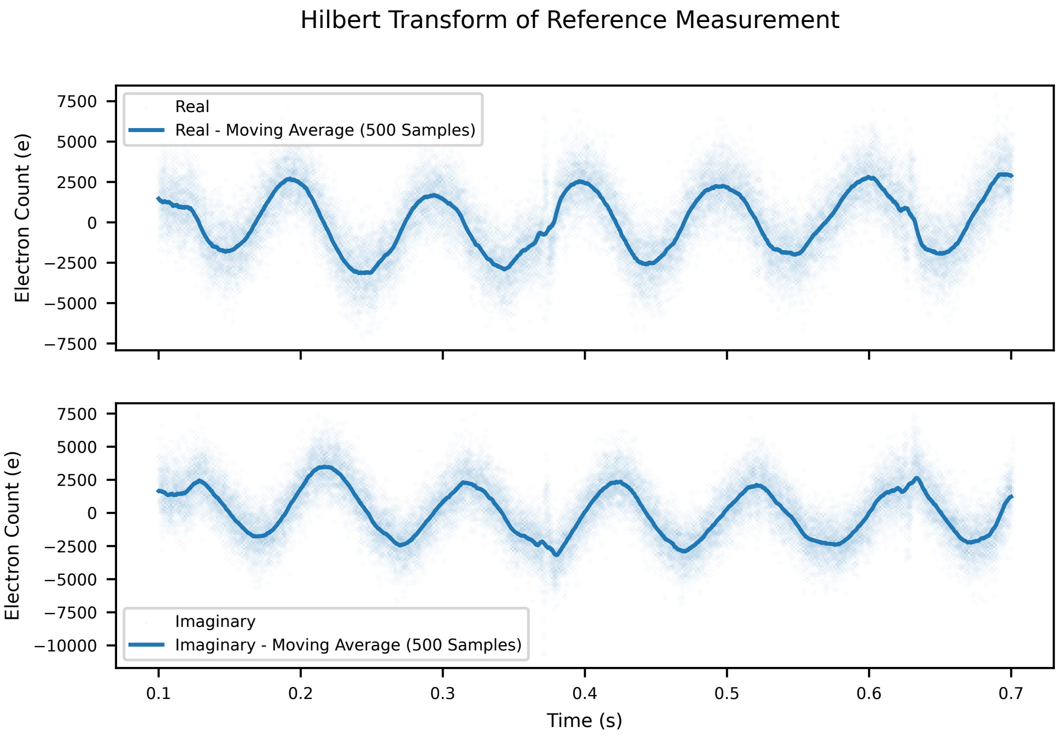

Supplementary Figure 19: **Hilbert Transformed CEP Signal:** The top and the bottom panel show the real and the imaginary component of the Hilbert transformed measurement data. Additionally the moving average is shown as solid blue lines.

To understand the phase-noise resulting from our measurement, we are using the Hilbert transformation to produce a complex valued signal from our data. The Hilbert transformation produces an analytical signal from a real valued one as produced by our measurement. From the analytical signal, as shown by the real and imaginary valued components in Fig. 19, we use the phasor representation $\tilde{S}(t) = A(t) \cdot e^{i\varphi(t)}$ to intuitively understand and plot the instantaneous phase $\varphi(t)$ of our measurement.

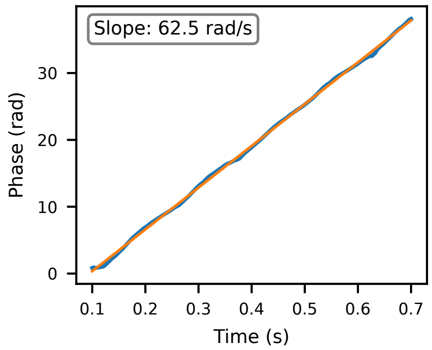

Supplementary Figure 20: **Time Series of the Phase:** Phase as a function of time extracted from the measurement data (blue) and a linear fit (orange) with a slope of 62.5 rad s^{-1}.

The retrieved phase $\varphi(t)$ is shown Fig. 20 with an additional linear fit. In the phase signal we can see the linear phase shift induced in the experiment, closely matching the experimentally induced phase-scan speed of 62.8 rad s^{-1}. By removing this linear phase movement we can estimate the rms phase noise in our experiment.

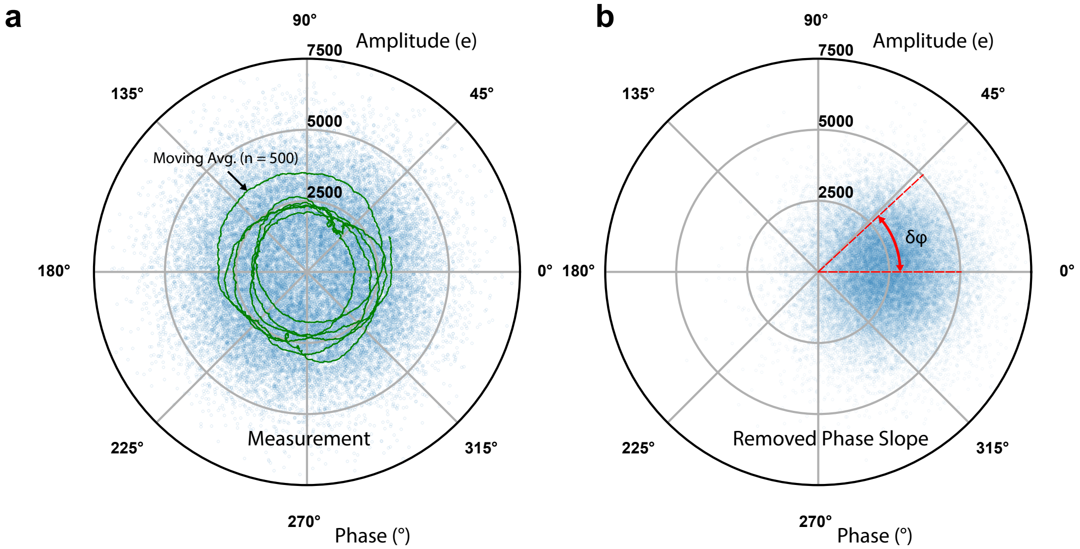

Supplementary Figure 21: **Synthetic Phasor Diagram: a**, Phasor representation of the Hilbert-transformed measurement data. The green line shows the data filtered by a moving average of n=500 to visualize the underlying phase movement. **b**, Phasor representation of the Hilbert-transformed measurement data with the removed phase slope.

For a more intuitive representation of our complex signal, we plot the phasor representation of the initial

measurement data in Fig. 21 a. This polar plot shows amplitude and phase of the signal and forms for the original case a donut distribution of the data points, as the phase of our signal rotates by 20π over 600 ms. To extract the rms phase noise we remove the linear phase movement, with the result shown in Fig. 21 b. From the distribution of $\varphi(t)$ the standard deviation $\delta\varphi$ is retrieved and yields a rms noise of 0.75 rad.

To put this result into perspective we calculated the contribution of 1100 e rms noise to an artificial sinusoidal signal of 10 Hz with an amplitude of 2370 e. This helps to understand the contribution of electronic noise to the total signal noise of 750 mrad rms, that was measured. Assuming we are dealing with uncorrelated noise sources, the total noise is expressed as the square-root over the sum of the individual contributions squared,

$$\delta\varphi_{\text{Total}} = \sqrt{\delta\varphi^2_{\text{Electr.}} + \delta\varphi^2_{\text{Shot}} + \delta\varphi^2_{\text{Laser}}}. \tag{7}$$

Considering that we have an estimate for the electronic noise contribution of 550 mrad rms, see Fig. 22 a, and a shot noise contribution of $\sqrt{2730} \approx 50$ e rms from the nanoantenna signal plus an additional charge background of 25000 e corresponding to shot noise of 160 e rms, we can estimate that the contribution of the laser is on the order of 480 mrad. This would include any contribution from the phase noise, which is on the order of 190 mrad rms measured at a bandwidth of 1 kHz. Further study will be necessary to establish a ground truth to the laser phase noise, as for example by using fast f-2f schemes as introduced by Guo et al. [17].

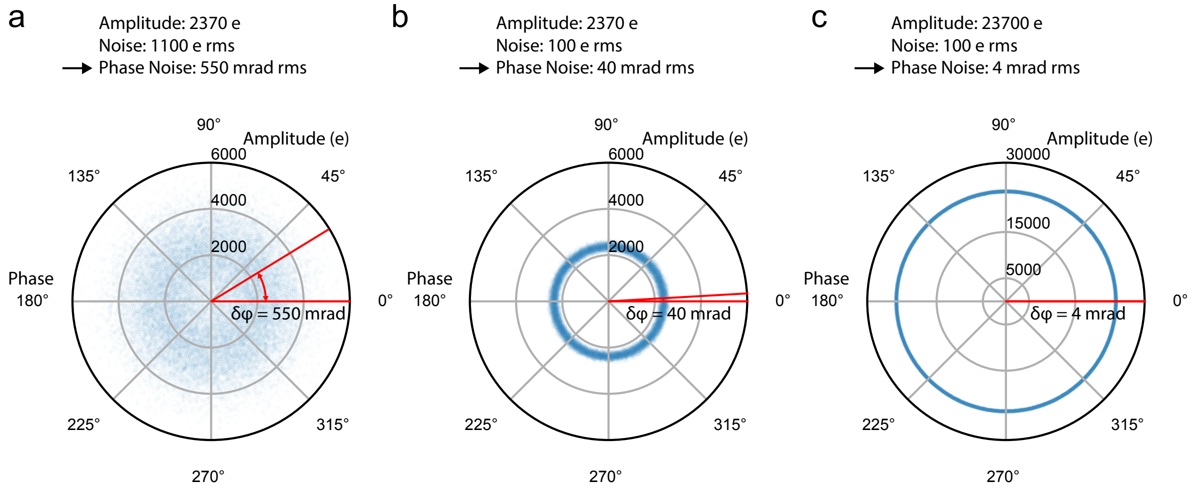

Supplementary Figure 22: **Comparison of different Phase Noise Levels: a,** Electronic noise (1100 e rms) contribution to the phase noise for a 10 Hz sinusoidal signal of amplitude 2370 e. **b** Electronic noise (100 e rms) contribution to the phase noise for a 10 Hz sinusoidal signal of amplitude 2370 e. **c** Electronic noise (100 e rms) contribution to the phase noise for a 10 Hz sinusoidal signal of amplitude 23700 e.

With this analysis we can also establish how specific improvements in electronic noise or signal amplitude translate into an improved phase noise. Considering electronic detection noise at 100 e rms, as would be the limit of the amplifier used in this manuscript, we expect a phase noise contribution of 40 mrad rms (see Fig. 22b). Furthermore, when implementing improvements in the device design or by increasing the network area, a 10-fold improvement in amplitude will result in an electronic noise contribution corresponding to around 4 mrad (see Fig. 22c). However, in this regime we would be predominantly shot-noise limited with 160 e rms, which would correspond to a phase noise of approximately 7 mrad. This analysis highlights the potential for using petahertz electronic devices to realize a phase detector that is highly sensitive, but is also extremely compact and allows for micrometer scale integration.

4.5. Larger Area Network

To verify the results measured in the main text we repeated the same measurements with an antenna network that measures 30 µm by 30 µm, which is substantially larger than the FWHM beam width of $\sim$ 21 µm. The other difference between these measurements is the use of a different detector for the single-shot pulse energy, which is, in this case, a commercial mercury cadmium telluride detector. This amounts to roughly

1000 antennas within the spatial FWHM contributing to the measured charge yield. A single-shot measurement is shown in Fig. 23. Identical to the main text, a clear CEP modulation is present in the measured data. Furthermore, also the same signatures of the piezo slip-stick motion are present in the data. In addition, we see a 3x larger background charge signal compared to the other measurement.

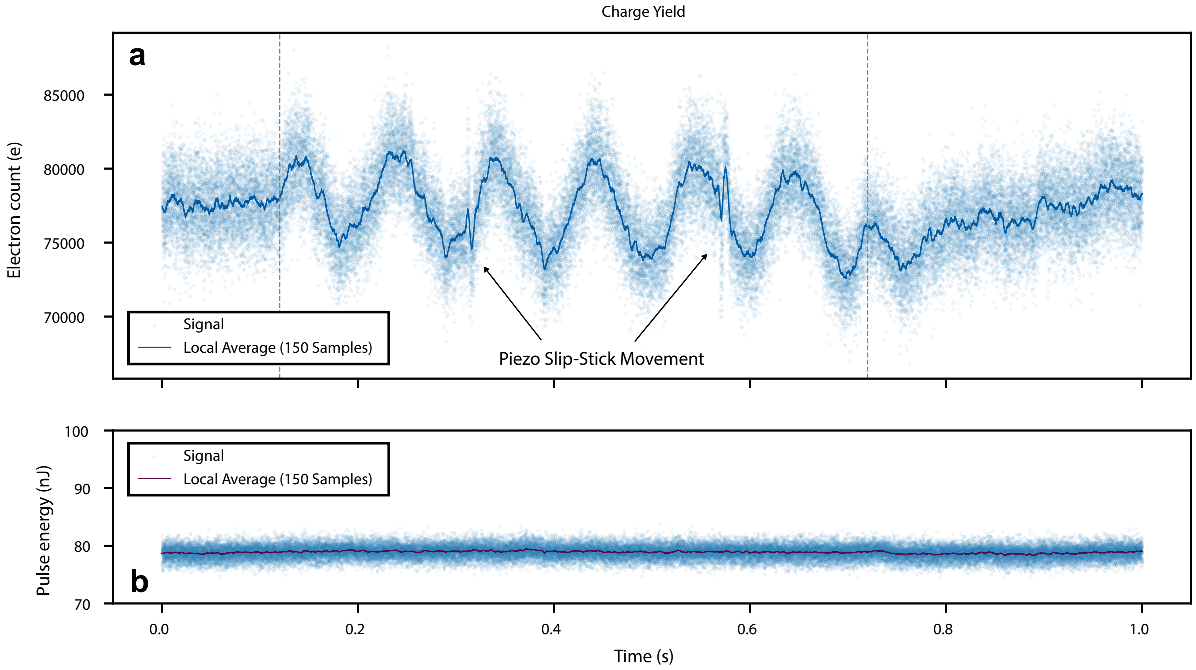

Supplementary Figure 23: **Single-shot charge readout:** A single dataset recording of 50 000 laser shots for the charge yield of the nanoantenna detector (**a**) and the laser energy recorded by the pyroelectric detector (**b**). The peak field of the incident laser pulse on the array is 1.6 V nm. From 120 ms to 720 ms the CE phase is linearly ramped over 6 cycles.

As in the main text, the same frequency analysis of the single-shot measurement is shown, which presents reproducible behavior. Aside from the clear CEP peak, we see in addition the same $1/f^{3/4}$ noise characteristic is present in the data. The narrow band noise peaks at $\sim$ 17 kHz in the electron amplitude are clearly discernible from noise. In conjunction with the higher background charge signal, we can strengthen the argument that the noise peaks are driven by high-frequency laser intensity changes predominantly modulating the background charge signal.

Analysing the scaling of the CEP peak as a function of the incident peak field (Fig. 25), we find in general similar behavior to Fig. 15. The Fowler-Nordheim fit results in a field-enhancement $g = 8$, very

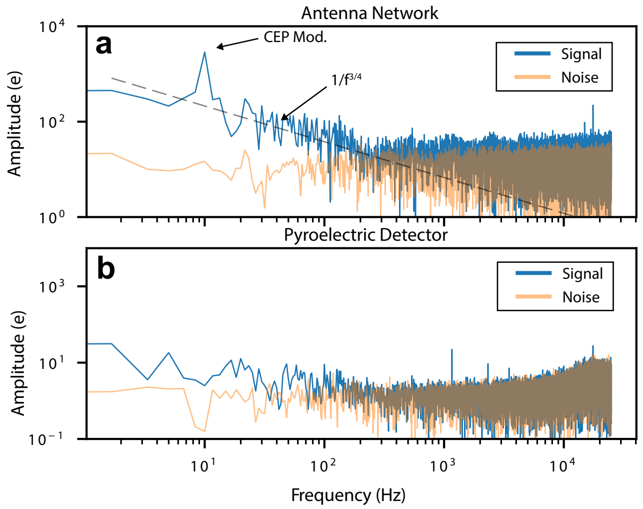

Supplementary Figure 24: **Frequency Domain of the single-shot data:** The respective data from Fig. 23 $t = 370\,\text{ms}$ to $t = 620\,\text{ms}$ is Fourier transformed and shown in charge amplitude as a function of frequency. For comparison, the electronic noise floor is shown in orange for both spectra. (**a**) the frequency-resolved signal of the nanoantenna network. (**b**) the frequency-resolved energy signal, as a function of pyroelectric charge yield.

close to the simulated field enhancement of 8.2. Furthermore, the prefactor $\alpha = 1647$ is almost identical to the one in Fig. 15 text with $\alpha' = 1517$, indicating that this array, despite the larger size, has a comparable amount of antennas contributing to the charge signal, as a is proportional to the number of antennas. Further measurements with different array sizes could map more precisely how many antennas are involved in the charge signal. The heuristic power law fit, $Q(E) = aE^n + c$, shows as well comparable behavior to the array discussed in the main text. The fit results are $a = 147$, $n = 7.65$, and $c = 13$, which in particular with the power law order n agrees very well with the main text where $n' = 7.85$ is measured. This shows that the measurements are in general of predictable behavior. Although the scaling law for low field strengths is not fully explained, models like the Yudin-Ivanov [15], could help to explain these scaling behaviors.

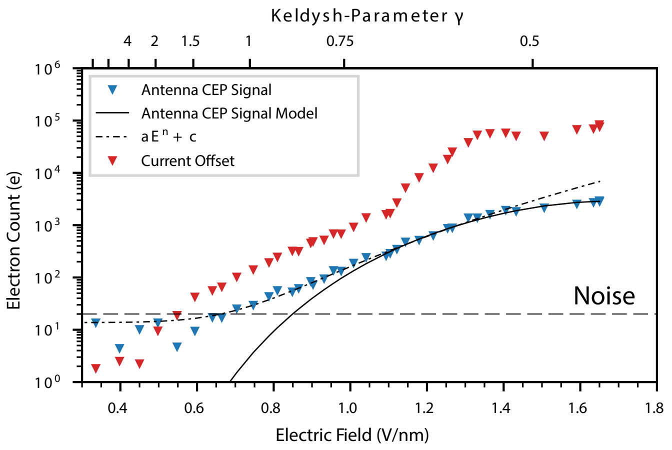

Supplementary Figure 25: **Charge yield scaling:** The CE phase modulation amplitude and the average charge yield are plotted as a function of the average peak field for the respective dataset. The CE phase signal is taken from the amplitude of the 10 Hz frequency component of the measurement data. The antenna CE phase signal model uses the model described by Eq. 3. Furthermore, a power law fit, $aE^n + c$ to the first 30 values is shown.

References

[1]S. V. Yalunin, M. Gulde, and C. Ropers, “Strong-field photoemission from surfaces: Theoretical approaches”, Physical Review B **84**, Number: 19, 195426 (2011).

[2]R. H. Fowler and L. Nordheim, “Electron Emission in Intense Electric Fields”, Royal Society of London Proceedings Series A **119**, 173–181 (1928).

[3]Y. Yang, M. Turchetti, P. Vasireddy, W. P. Putnam, O. Karnbach, A. Nardi, F. X. Kärtner, K. K. Berggren, and P. D. Keathley, “Light phase detection with on-chip petahertz electronic networks”, Nature Communications **11**, 3407 (2020).

[4]A. D. Rakić, “Algorithm for the determination of intrinsic optical constants of metal films: application to aluminum”, EN, Applied Optics **34**, Publisher: Optica Publishing Group, 4755–4767 (1995).

[5]M. R. Bionta, F. Ritzkowsky, M. Turchetti, Y. Yang, D. Cattozzo Mor, W. P. Putnam, F. X. Kärtner, K. K. Berggren, and P. D. Keathley, “On-chip sampling of optical fields with attosecond resolution”, Nature Photonics **15**, 456–460 (2021).

[6]T. Rybka, M. Ludwig, M. F. Schmalz, V. Knittel, D. Brida, and A. Leitenstorfer, “Sub-cycle optical phase control of nanotunnelling in the single-electron regime”, Nature Photonics **10**, 667–670 (2016).

[7]M. Ludwig, G. Aguirregabiria, F. Ritzkowsky, T. Rybka, D. C. Marinica, J. Aizpurua, A. G. Borisov, A. Leitenstorfer, and D. Brida, “Sub-femtosecond electron transport in a nanoscale gap”, en, Nature Physics **16**, Number: 3 Publisher: Nature Publishing Group, 341–345 (2020).

[8]W. P. Putnam, R. G. Hobbs, P. D. Keathley, K. K. Berggren, and F. X. Kärtner, “Optical-field-controlled photoemission from plasmonic nanoparticles”, en, Nature Physics **13**, Number: 4, 335–339 (2017).

[9]F. Ritzkowsky, E. Bebeti, G. M. Rossi, R. E. Mainz, H. Suchowski, H. Cankaya, and F. Kaertner, “Passively CEP stable sub-2-cycle source in the mid-infrared by adiabatic difference frequency generation”, EN, Optics Letters, Publisher: Optica Publishing Group, `10.1364/OL.485610` (2023).

[10]J. R. Birge, R. Ell, and F. X. Kärtner, “Two-dimensional spectral shearing interferometry for few-cycle pulse characterization”, EN, Optics Letters **31**, 2063–2065 (2006).

[11]J. R. Birge, H. M. Crespo, and F. X. Kärtner, “Theory and design of two-dimensional spectral shearing interferometry for few-cycle pulse measurement”, JOSA B **27**, 1165–1173 (2010).

[12]I. E. Gordon, L. S. Rothman, R. J. Hargreaves, R. Hashemi, E. V. Karlovets, F. M. Skinner, E. K. Conway, C. Hill, R. V. Kochanov, Y. Tan, P. Wcisło, A. A. Finenko, K. Nelson, P. F. Bernath, M. Birk, V. Boudon, A. Campargue, K. V. Chance, A. Coustenis, B. J. Drouin, J. .-. Flaud, R. R. Gamache, J. T. Hodges, D. Jacquemart, E. J. Mlawer, A. V. Nikitin, V. I. Perevalov, M. Rotger, J. Tennyson, G. C. Toon, H. Tran, V. G. Tyuterev, E. M. Adkins, A. Baker, A. Barbe, E. Canè, A. G. Császár, A. Dudaryonok, O. Egorov, A. J. Fleisher, H. Fleurbaey, A. Foltynowicz, T. Furtenbacher, J. J. Harrison, J. .-. Hartmann, V. .-. Horneman, X. Huang, T. Karman, J. Karns, S. Kassi, I. Kleiner, V. Kofman, F. Kwabia–Tchana, N. N. Lavrentieva, T. J. Lee, D. A. Long, A. A. Lukashevskaya, O. M. Lyulin, V. Y. Makhnev, W. Matt, S. T. Massie, M. Melosso, S. N. Mikhailenko, D. Mondelain, H. S. P. Müller, O. V. Naumenko, A. Perrin, O. L. Polyansky, E. Raddaoui, P. L. Raston, Z. D. Reed, M. Rey, C. Richard, R. Tóbiás, I. Sadiek, D. W. Schwenke, E. Starikova, K. Sung, F. Tamassia, S. A. Tashkun, J. Vander Auwera, I. A. Vasilenko, A. A. Vigasin, G. L. Villanueva, B. Vispoel, G. Wagner, A. Yachmenev, and S. N. Yurchenko, "The HITRAN2020 molecular spectroscopic database", Journal of Quantitative Spectroscopy and Radiative Transfer **277**, 107949 (2022).

[13]O. Oliaei, "Noise analysis of correlated double sampling SC integrators with a hold capacitor", IEEE Transactions on Circuits and Systems I: Fundamental Theory and Applications **50**, Number: 9 Conference Name: IEEE Transactions on Circuits and Systems I: Fundamental Theory and Applications, 1198–1202 (2003).

[14]H. Wey and W. Guggenbuhl, "Noise transfer characteristics of a correlated double sampling circuit", IEEE Transactions on Circuits and Systems **33**, Number: 10 Conference Name: IEEE Transactions on Circuits and Systems, 1028–1030 (1986).

[15]G. L. Yudin and M. Y. Ivanov, "Nonadiabatic tunnel ionization: Looking inside a laser cycle", Physical Review A **64**, Number: 1, 013409 (2001).

[16]L. Shi, I. Babushkin, A. Husakou, O. Melchert, B. Frank, J. Yi, G. Wetzel, A. Demircan, C. Lienau, H. Giessen, M. Ivanov, U. Morgner, and M. Kovacev, "Femtosecond Field-Driven On-Chip Unidirectional Electronic Currents in Nonadiabatic Tunneling Regime", en, Laser & Photonics Reviews **15**, Number: 8 _eprint: https://onlinelibrary.wiley.com/doi/pdf/10.1002/lpor.202000475, 2000475 (2021).

[17]C. Guo, M. Miranda, A.-K. Raab, A.-L. Viotti, P. T. Guerreiro, R. Romero, H. Crespo, A. L'Huillier, and C. L. Arnold, "Single-shot, high-repetition rate carrier-envelope-phase detection of ultrashort laser pulses", EN, Optics Letters **48**, Publisher: Optica Publishing Group, 5431–5434 (2023).
